# Supplementary figures and images for: Caregiver experiences of an integrative patient-centered digital health application for pediatric type 1 diabetes care: Findings from a pilot clinical trial
Source: PLOS Digit Health. 2025 Oct 31;4(10):e0000861. doi: 10.1371/journal.pdig.0000861 (PMC12578157; doi:10.1371/journal.pdig.0000861)

Supporting Information 1. Clinic Visit Preparation Form

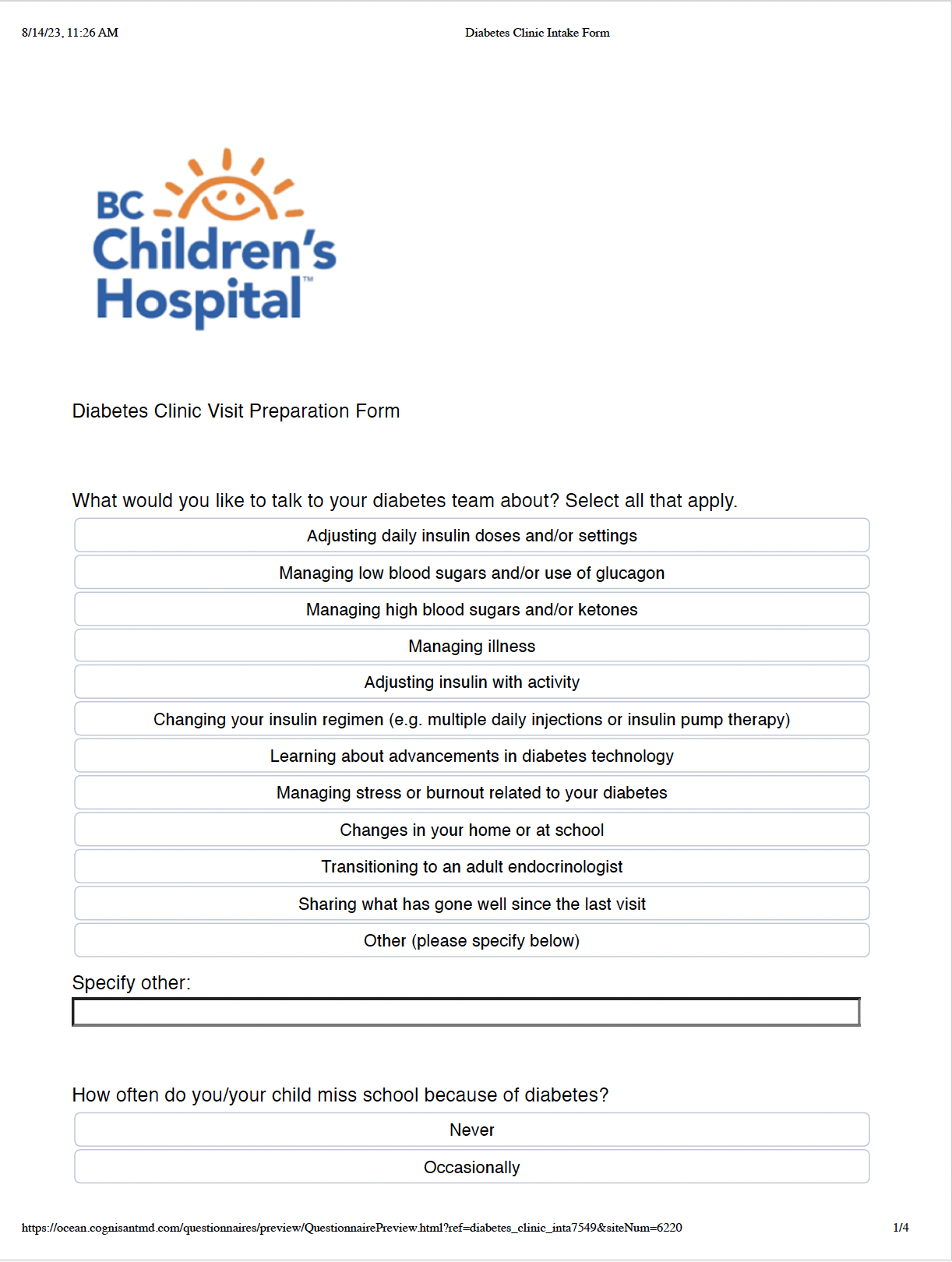


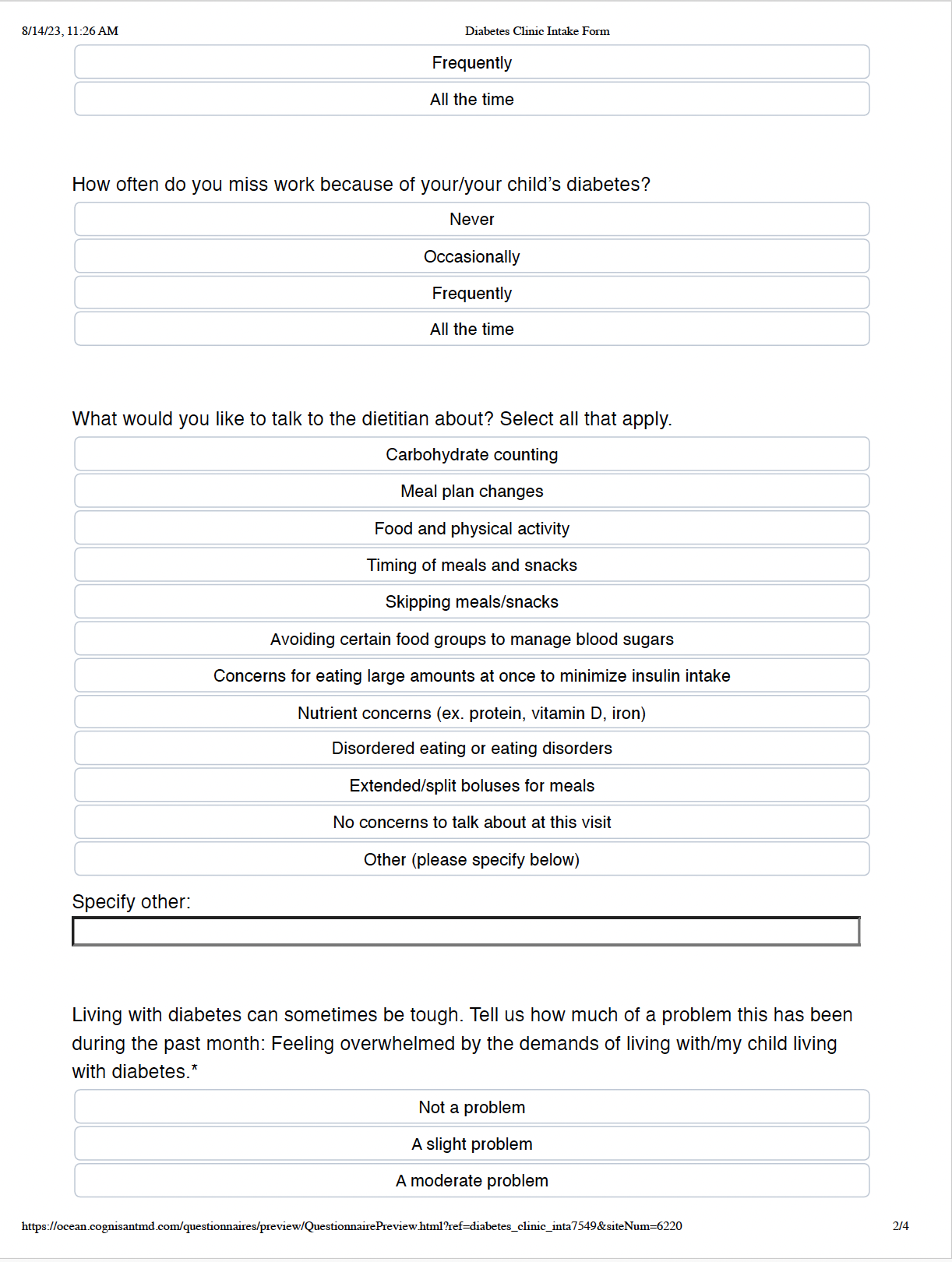

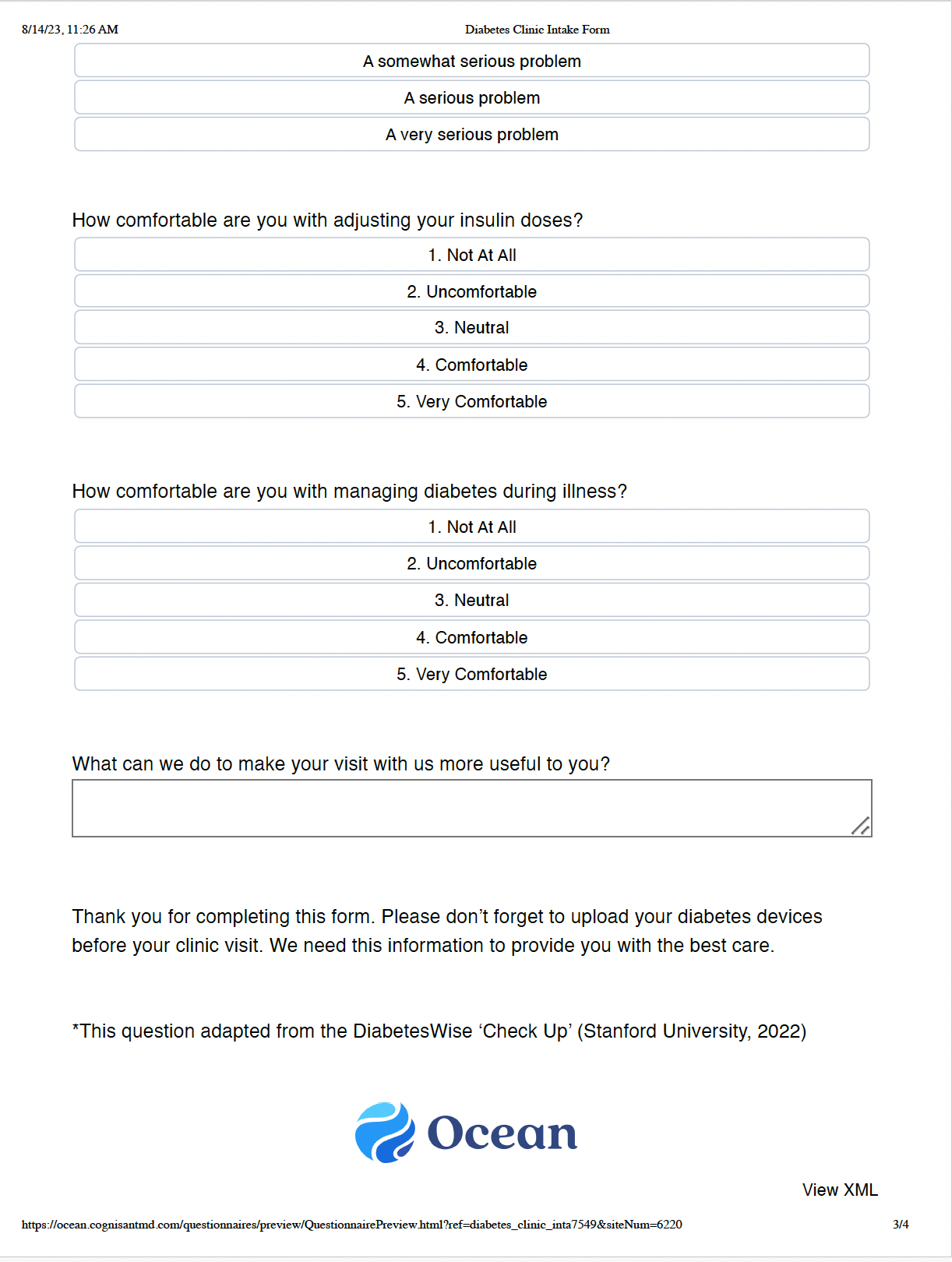

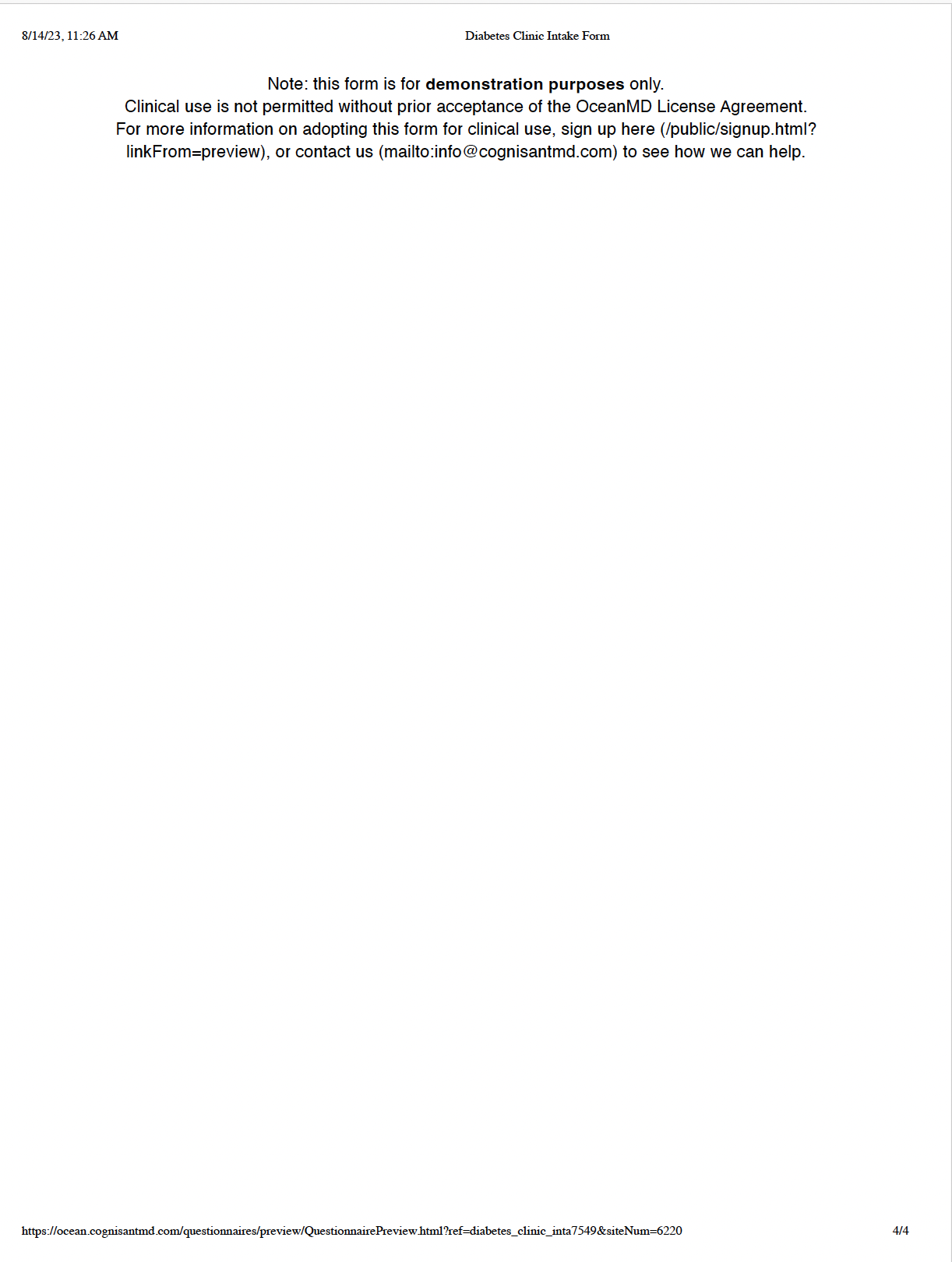

Supplement: S1 File — (DOCX) [file pdig.0000861.s001.docx]

Supporting Information 4. Participant Flow


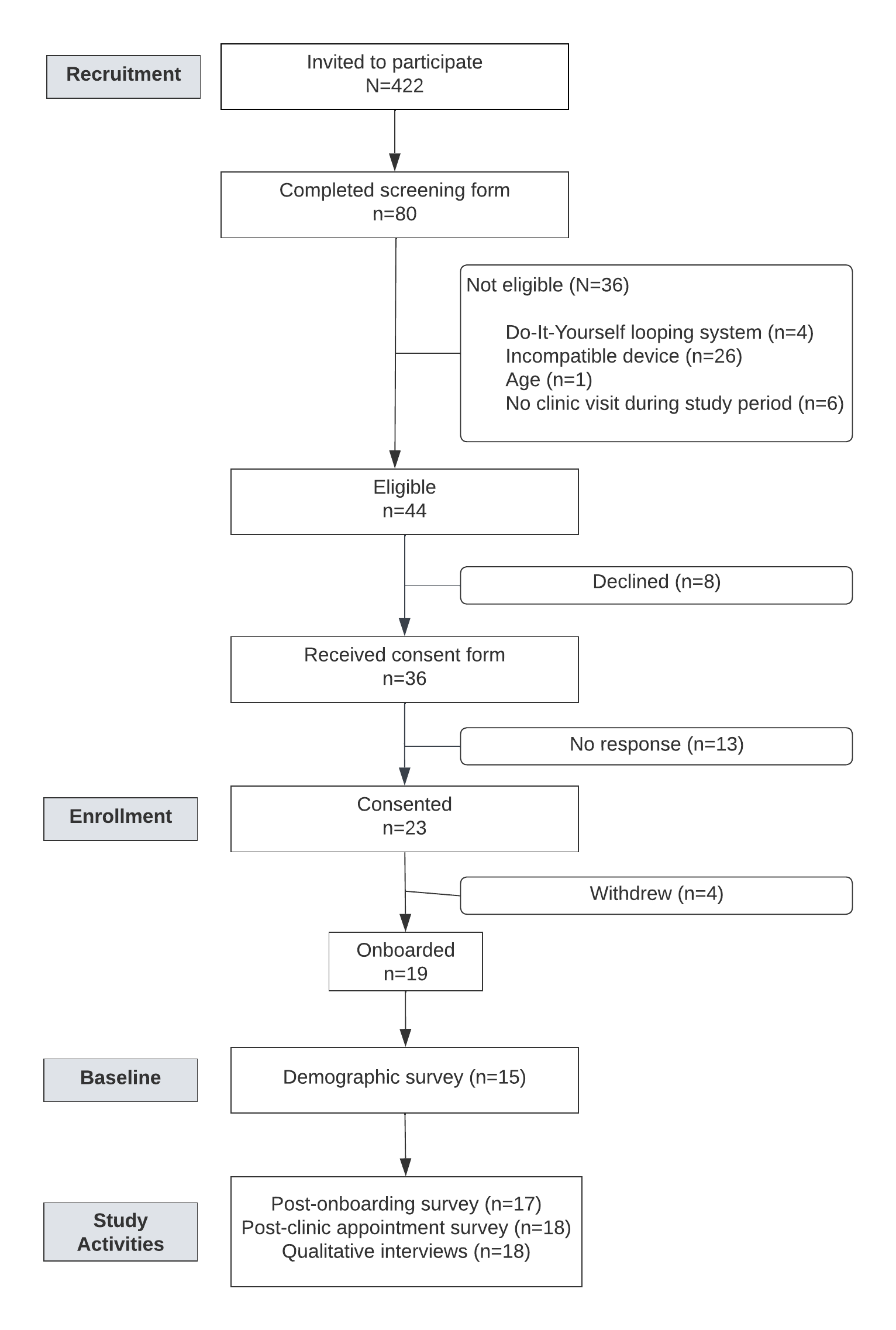

Supplement: S4 File — (DOCX) [file pdig.0000861.s004.docx]
