# Supplementary material for: Caregiver experiences of an integrative patient-centered digital health application for pediatric type 1 diabetes care: Findings from a pilot clinical trial
Source: PLOS Digit Health. 2025 Oct 31;4(10):e0000861. doi: 10.1371/journal.pdig.0000861 (PMC12578157; doi:10.1371/journal.pdig.0000861)
Supplement: Attachment — Submitted filename: Response to Reviewers.docx [file pdig.0000861.s007.docx]

**Response to Reviewers**

**Manuscript ID: PDIG-D-25-00235**

**Manuscript Title: Caregiver experiences of an integrative patient-centered digital health application for pediatric type 1 diabetes care: findings from a pilot clinical trial**

Reviewer #1: I read with great interest the manuscript "Caregiver experiences of an integrative patient-centered digital health application for pediatric type 1 diabetes care: findings from a pilot clinical trial" by Amed et al. I truly enjoy the ambitious approach to both developing and carefully testing a joint agnostic platform for diabetes data which is truly a need in clinical diabetes management. After reading the manuscript I only have a few minor questions that I think could be worth adressing:

Response: We thank the reviewer for their comments.
1. Could you please provide descriptive data of the patients included, i.e. age, age at diagnosis etc and also previous use of CGM and/or insulin pump

Response: Descriptive data of patients is included in-text the manuscript under the results section within the sub header ‘Demographic Characteristics.’ We have added additional descriptive data:

Among the 19 caregivers who participated in the study, the median (interquartile range [IQR]) age was 45 [39, 46] years. Most caregivers were white (n=10/15, 68%) and identified as mothers (14/19, 74%) and were married (11/15, 73%). The median age of their children was 10 [8, 13] years and were living with diabetes for 4.8 (±3.7) years at the time of data collection. Most children (n=18) used CGM to monitor their blood glucose while one child used a glucometer. Insulin was administered either by insulin pump (n=10) or through multiple daily injections (n=9). [page 9, paragraph 2, lines 156-159]

2. Could you please provide descriptive data on the HCPs and their previous experience from working with CGM data and other platforms.

Response: Given the small sample size of participating HCPs, we did not collect data describing their socio-demographics. We have added to the manuscript, HCP’s previous experience viewing CGM and pump data:

A total of 11 HCP agreed to participate in the pilot study and completed post-clinic surveys to gather their perceptions on the usefulness of Careteam’s web-based application before and during clinical visits with study participants. At baseline, HCP predominantly had experience using Dexcom Clarity and Glooko to visualize CGM and pump data. [page 15, paragraph 1, lines 202 and 203]
3. Did the HCPs or caregivers have previous experience from other platforms such as Tidepool and in what way does your MVP platform differ from such platforms.

Response: Tidepool is not a commonly used platform in the BC Children’s Hospital Diabetes Clinic and therefore, HCPs and caregivers had limited to no experience using Tidepool. To our knowledge, our MVP platform is different from platforms like Tidepool in that in addition to patients and HCPs viewing the same data from CGM and pumps, patients can complete forms between clinic visits, view HCP recommendations, enter multiple daily injection doses, and navigate to educational resources. Different from platforms like Tidepool, our MVP provides a ‘one-stop’ shop where patients can access all of the information they need, when they need it.
