# Supplementary material for: Caregiver experiences of an integrative patient-centered digital health application for pediatric type 1 diabetes care: Findings from a pilot clinical trial
Source: PLOS Digit Health. 2025 Oct 31;4(10):e0000861. doi: 10.1371/journal.pdig.0000861 (PMC12578157; doi:10.1371/journal.pdig.0000861)
Supplement: Attachment — Submitted filename: Response_to_Reviewers_auresp_2.docx [file pdig.0000861.s008.docx]

**Response to Reviewers**

**Manuscript ID: PDIG-D-25-00235R2**

**Manuscript Title: Caregiver experiences of an integrative patient-centered digital health application for pediatric type 1 diabetes care: findings from a pilot clinical trial**

**Reviewer #2 comments:**

*Thank you for the opportunity to review this insightful manuscript. I commend your team for developing, deploying, and evaluating this patient-centered digital health platform for pediatric T1D population. This real-world pilot study is methodologically sound, and your mixed-methods approach (blending quantitative survey data with rich qualitative insights) offers a compelling view into caregiver experiences, perceived value, and implementation challenges. It is evident that this work is the result of a thoughtful, collaborative, and multidisciplinary effort.

Your manuscript is well-written, engaging, and clearly addresses an important gap in digital health for chronic disease management, particularly in pediatrics, where caregiver engagement is vital. The emphasis on co-design with end users, iterative feedback integration, and usability testing reflects strong alignment with human-centered design principles. Caregivers' voices come through clearly in your qualitative findings, and the themes you've presented (convenient connection, usage drives value, and hope for future benefit) are both meaningful and actionable.

That said, I'd like to offer several suggestions that may help strengthen the paper further and increase its utility for readers interested in implementation science and digital health innovation:*

Response: We thank the reviewer for their comments.

*1. While the manuscript acknowledges the sample's demographic limitations, a more detailed reflection on how future phases might ensure greater diversity (ethnic, socioeconomic, technological literacy) would be valuable. This is especially important in digital health, where equity and access are persistent challenges.*

Response:

We thank the reviewer for this important observation. As we move on to evaluating this platform in a quasi-experimental trial at BC Children’s Hospital, exploring its implementation and assessing its effectiveness, we are expanding our recruitment strategy.

- We will strive to recruit a sample whose ethnic distribution reflects the prevalence of type 1 diabetes in the Canadian population (approximately 70% Caucasian, 15% South Asian, and 15% other).
- Our recruitment strategy for the trial includes both in-person and electronic approaches to ensure we reach participants from all socioeconomic backgrounds.
- In terms of technological literacy, we plan to administer the eHealth Literacy Scale (eHEALS) (score 8-40) to capture caregivers perceived ability to locate, evaluate and apply online health information. Outcome analyses will be stratified by eHEALS tertile to understand how technology was adopted and what challenges the users might have confronted.

We have added a statement reflecting this under the ‘Future Directions’ section in our manuscript. (page number: 25; line numbers: 402-407)

*2. The focus on caregivers is understandable for a pediatric population, but a discussion on why children or adolescents weren't interviewed, and whether they will be involved in future evaluations, would enrich the perspective.*

Response:

We appreciate the reviewer’s suggestion to include a rationale for focusing on caregivers in this pilot study. Given the small scale of the study, we recruited caregivers as the primary respondents. However, we fully agree that incorporating youth voices is critical to understanding engagement and acceptability, particularly among adolescents with more autonomous self-management. This limitation of our study is referenced in the Strengths and Limitations section (page number 25, line numbers 396-398). In our next phase, we plan to enrol (1) at least 30 % youth participants aged 15-17 who are primary decision-makers in their diabetes care, and (2) a broader cohort of younger children whose parents remain the primary managers. This will also allow us to stratify findings by type of user and explore potential differences in needs, preferences, and digital health uptake across age groups.

We have added a statement reflecting this under the ‘Future Directions’ section in our manuscript. (page number: 25; line numbers: 407-409)

*3. Including even brief commentary on technical usability like login issues, sync failures, or onboarding difficulties, would offer readers a fuller picture of real-world feasibility.*

Response:

We thank the reviewer for this comment. While we acknowledge that technical usability data is key for mHealth studies, unfortunately, we were unable to obtain high quality user data from the provider of the frontend application. We acknowledge this as a limitation. The upcoming trial will include detailed technical usability data to address this gap.

We have added a statement reflecting this in our manuscript. (page number: 25; line numbers: 400-401)

*4. Your paper would benefit from a clearer breakdown of how different healthcare provider roles (e.g., endocrinologists vs. nurses vs. dietitians) engaged with the platform and how their workflows may have been affected.*

Response:

We agree that role-specific analysis of provider engagement would strengthen the paper. However, the sample size in our study was too small to support meaningful stratification by provider type (e.g., endocrinologist, nurse, dietitian). We have noted this as a limitation and plan to capture and analyse role-specific engagement and workflow impact in the upcoming trial, where a larger and more diverse provider sample will allow for meaningful comparison across disciplines.

*5. Given that most caregivers reported using the app monthly or less, it would be helpful to reflect on how the platform might maintain engagement over time, especially after the novelty of a pilot wears off.*

Response:

Thank you, we appreciate this observation. Sustained user engagement is closely tied to perceived value, and we recognise that novelty alone is not sufficient to maintain usage over time. In the next phase, we are working on continuously iterating the platform based on user feedback to enhance its relevance and usefulness; we see this important step as a primary driver of engagement. Additional strategies such as technical support, a detailed change management strategy and facilitated onboarding are also planned for implementation. We also plan on evaluating these implementation strategies systematically as part of the trial to understand their impact on long-term engagement.

*6. While the study is practical and applied, there is little engagement with behavioral health or technology adoption theory (e.g., Technology Acceptance Model, Health Belief Model, COM-B). Consider suggesting a theoretical frame to contextualize caregiver behaviors and attitudes, which may help guide future iterations or hypothesis-driven trials.*

Response:

We appreciate this valuable suggestion. While we did not explicitly apply behavioural or technology adoption frameworks in the current pilot, our future work will be guided by established models, including the Technology Acceptance Model and behavioural frameworks such as COM-B, to better understand user engagement and diabetes behaviour change.


*7. The architecture's mention of (Fast Healthcare Interoperability Resources) suggests alignment with interoperability standards, which is appreciated. However, only minimal technical detail is provided. Consider expanding slightly on how FHIR was implemented in this context, for example, whether it enabled integration with EMRs, how data security or identity management was handled, and whether institutional or technical challenges affected scaling the platform beyond BCCH.*

Response:

We thank the reviewer for highlighting the importance of providing more technical detail. A separate manuscript focused on the platform’s technical architecture, including FHIR implementation, data security, and identity management is currently under review with JMIR Pediatrics and Parenting (<https://preprints.jmir.org/preprint/70061>). To maintain focus in this paper, we have referenced that work in the paper. Additionally, for your reference, we have pasted (see below) from that paper, a brief summary of the key technical elements relevant to FHIR use and interoperability. We have also ensured to cite the preprint of this paper in the manuscript.


“The TrustSphere platform is designed as a service-oriented architecture, where components and modules communicate via application programming interfaces (APIs) and web services. Each component focuses on a specific aspect of the functional layers and becomes an interchangeable part of the whole. Modules initiate and complete their tasks synchronously for real-time data or asynchronously for batch data. Data exchange between the modules occurs using RESTful services and the Fast Healthcare Interoperability Resources (FHIR) exchange standards. The system utilizes TrustSphere-tailored components from commercial partners, including Federated Privacy Exchange and Authorization layer (FPX), and a Care Hub application developed by IDENTOS (Toronto, ON, Canada), and a collaborative patient and HCP platform developed by Careteam Technologies (Vancouver, BC, Canada). As an Authorization technology platform, FPX provides digital authentication, authorization, and governance capabilities on the foundation of OAuth 2.0 and can support User Managed Access protocols as well as federated identity including with the BC Government identity and Interac (Toronto, ON) Verified identification services. The internal components of FPX modules are developed in Java programming language. TrustSphere acquires diabetes device PGHD using restful APIs provided by Glooko (Mountain View, CA, USA). The acquired data are transformed into Health Level 7 (HL7) data exchange standard FHIR resources by a FHIR Transformation Engine, developed by Smile Digital Health (Toronto, ON), and are stored in a NoSQL database. Careteam’s platform, developed in PHP, was used to visualize the collected data for patients, families, and HCPs.”

*8. The feedback on the consent/assent process (e.g., length, transparency) is valuable. It would be great to see how your team plans to iterate on this process, especially as you consider broader rollout.*

Response:

We appreciate the reviewer’s feedback on the consent and assent process. At this time, we are required to follow the REB-approved templates, including the BC Common Clinical Informed Consent Form and the standard assent forms provided by the REB. While we acknowledge that these forms can be lengthy, any modifications must align with current institutional guidelines. We understand that efforts are underway to revise and simplify these templates, and once a shorter version is approved, we will adopt it for future phases.

*Despite these suggestions, I found the manuscript to be a thoughtful and meaningful contribution. It's encouraging to see platforms designed with and for caregivers and healthcare teams that aim to improve communication, data visibility, and shared decision-making. Your findings not only provide a foundation for further platform development, but also offer useful insights to the broader digital health community working toward person- and family-centered care.

Thank you again for the opportunity to review this important work. I hope my comments are helpful as you finalize your manuscript, and I look forward to seeing the platform's evolution and continued impact in future studies.*
